# The JAK1/2 Inhibitor Baricitinib Ameliorates Neuroinflammation and Symptoms in an Animal Model of Essential Tremor

**DOI:** 10.1002/prp2.70299

**Published:** 2026-07-10

**Authors:** Mehran Ilaghi, Fatemeh Shahsavari, Monavareh Soti, Sina Elyasifar, Leila Bagherzadeh, Zeynab Pirmoradi, Mohammad Shabani

**Affiliations:** ^1^ Neuroscience Research Center, Institute of Neuropharmacology Kerman University of Medical Sciences Kerman Iran; ^2^ Department of Physical Medicine and Rehabilitation Northwestern University Feinberg School of Medicine Chicago Illinois USA; ^3^ Neuroscience Research Center Torbat Heydariyeh University of Medical Sciences Torbat Heydariyeh Iran

**Keywords:** baricitinib, cerebellum, essential tremor, harmaline, JAK inhibitor, neuroinflammation

## Abstract

Essential tremor (ET) is one of the most prevalent adult‐onset movement disorders, which significantly impairs quality of life. So far, there are few therapeutic options with limited efficacy. This study investigated the therapeutic potential of baricitinib, a selective JAK1/2 inhibitor, in the harmaline‐induced model of ET. Male Swiss mice were randomly assigned to five groups (*n* = 8): control, harmaline, and harmaline plus baricitinib at 10, 20, or 50 mg/kg doses. ET was induced using harmaline (10 mg/kg, i.p.). Baricitinib was administered orally daily for 14 days. Assessments included behavioral tests and cerebellar gene expression analysis of inflammatory markers (TNF‐α, IL‐6, NF‐κB), neurotrophic factors (BDNF), ET‐related targets (Lingo‐1, NMDA receptor), and histopathological evaluation. Baricitinib reduced harmaline‐induced tremor severity. The drug also significantly ameliorated anxiety‐like behaviors in elevated plus maze and open field tests, while reducing depressive‐like behavior in the tail suspension test. Passive avoidance outcomes changed in a direction suggestive of improved retention; however, the high‐dose findings should be interpreted cautiously because 50 mg/kg was associated with motor coordination impairment. Molecular analyses revealed baricitinib completely normalized cerebellar pro‐inflammatory cytokines TNF‐α and IL‐6 and suppressed NF‐κB expression. The drug restored BDNF levels and reduced Lingo‐1 expression. Histopathological examination also suggested reduced Purkinje cell degeneration, with 20 and 50 mg/kg doses reducing Purkinje cell degeneration to control levels. Baricitinib reduced tremor and affective behaviors in a harmaline‐induced ET model, though gait abnormalities persisted. Cognitive benefits at lower doses were observed, with cautious interpretation required for the high dose due to motor effects. Molecular and histological analyses supported anti‐inflammatory and neuroprotective effects, indicating selective benefits via neuroinflammatory modulation.

## Graphical abstract

In a harmaline‐induced essential tremor mouse model, baricitinib attenuated tremor severity and affective‐like abnormalities, suppressed cerebellar TNF‐α, IL‐6, NF‐κB, and Lingo‐1 expression, and showed supportive Purkinje‐cell morphological preservation. Benefits were selective, as gait widening persisted and high‐dose motor impairment requires caution.

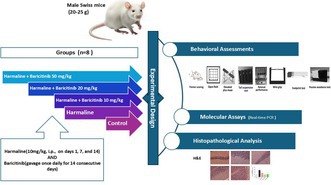

## 1 Introduction

Essential tremor (ET) is one of the most prevalent adult‐onset movement disorders, affecting approximately 0.9% of the global population, with prevalence rates exceeding 4% in individuals over 65 years of age [1, 2]. Characterized by involuntary rhythmic oscillations of body parts, particularly during postural maintenance and voluntary movements, ET significantly impairs patients' quality of life and functional independence [3]. Despite its high prevalence and substantial socioeconomic burden, the underlying pathophysiology of ET remains incompletely understood, and current therapeutic options are limited in efficacy and often associated with significant side effects [4].

The cerebellum, particularly the olivocerebellar pathway, has been identified as the primary anatomical substrate underlying ET pathophysiology, making it a critical target for therapeutic intervention [5]. The harmaline‐induced tremor model has emerged as the most widely validated experimental paradigm for studying ET, as it recapitulates many key features of the human condition [6]. Harmaline, a β‐carboline alkaloid, induces rhythmic tremor by causing synchronized oscillatory firing of inferior olivary neurons, which project climbing fibers to cerebellar Purkinje cells. This results in characteristic complex spike activity patterns that closely mirror those observed in ET patients [6]. The model's translational validity is further supported by its responsiveness to ET therapeutics and its ability to reproduce the cerebellar pathology associated with the disorder [7, 8].

Emerging evidence suggests that neuroinflammation plays a crucial role in the pathogenesis and progression of ET. Postmortem studies have revealed significant structural abnormalities in the cerebellum of ET patients, including an increase in Purkinje cell axonal swellings and substantial neuronal loss compared to healthy controls [9, 10]. These pathological changes are accompanied by increased levels of pro‐inflammatory cytokines [11], suggesting that inflammatory processes contribute to cerebellar dysfunction in ET. Additional molecular targets implicated in ET pathophysiology include Leucine‐rich repeat and immunoglobulin‐like domain‐containing protein 1 (Lingo‐1) and N‐methyl‐D‐aspartate (NMDA) receptors. Lingo‐1, a negative regulator of myelination and synaptic plasticity, is significantly upregulated in ET patients' cerebellar tissue [12, 13]. Concurrently, alterations in NMDA receptor expression and function contribute to abnormal glutamatergic signaling within olivocerebellar circuits, potentially exacerbating tremor generation and cerebellar excitotoxicity [6, 14].

The Janus kinase/signal transducer and activator of transcription (JAK/STAT) signaling pathway represents a critical mediator of inflammatory responses in the central nervous system (CNS). JAK proteins, including JAK1, JAK2, JAK3, and TYK2, form dimeric complexes that phosphorylate STAT proteins upon cytokine receptor activation, leading to transcriptional upregulation of inflammatory mediators [15, 16]. Dysregulation of JAK/STAT signaling has been implicated in various neurological disorders, including multiple sclerosis, Alzheimer's disease, and amyotrophic lateral sclerosis, highlighting its potential as a therapeutic target for neuroinflammatory conditions [17, 18].

Baricitinib, an oral selective inhibitor of JAK1 and JAK2, has demonstrated remarkable efficacy in treating autoimmune and inflammatory disorders, including rheumatoid arthritis, alopecia areata, and systemic lupus erythematosus [19, 20, 21]. The drug's mechanism of action involves competitive inhibition of ATP binding to JAK1 and JAK2, thereby preventing STAT phosphorylation and subsequent inflammatory gene transcription [20]. Recent preclinical studies have shown that baricitinib can effectively cross the blood–brain barrier and exert neuroprotective effects in models of neuroinflammation and neurodegeneration [22, 23].

Given the established role of neuroinflammation in ET pathogenesis and the anti‐inflammatory properties of baricitinib, we hypothesized that JAK1/2 inhibition could ameliorate tremor symptoms and associated cerebellar pathology in the harmaline model. Furthermore, considering the complex interplay between inflammation, oxidative stress, and neuronal dysfunction in ET, we anticipated that baricitinib treatment might also improve cognitive and affective symptoms commonly observed in ET patients.

## 2 Methods

### 2.1 Animals

Male Swiss mice weighing 20–25 g were obtained from Kerman University of Medical Sciences and housed in groups of 8 in standard cages for 2 weeks under a 12‐h light/dark cycle with free access to food and water. All experimental procedures were conducted in accordance with the Guidelines for the Care and Use of Laboratory Animals and approved by the Institutional Animal Care and Use Committee of Kerman University of Medical Sciences.

### 2.2 Experimental Design

Animals were randomly assigned to five experimental groups (*n* = 8 per group) as follows:

(i) Control, (ii) Harmaline, (iii) Harmaline + Baricitinib 10 mg/kg, (iv) Harmaline + Baricitinib 20 mg/kg, and (v) Harmaline + Baricitinib 50 mg/kg.

**FIGURE 1:**
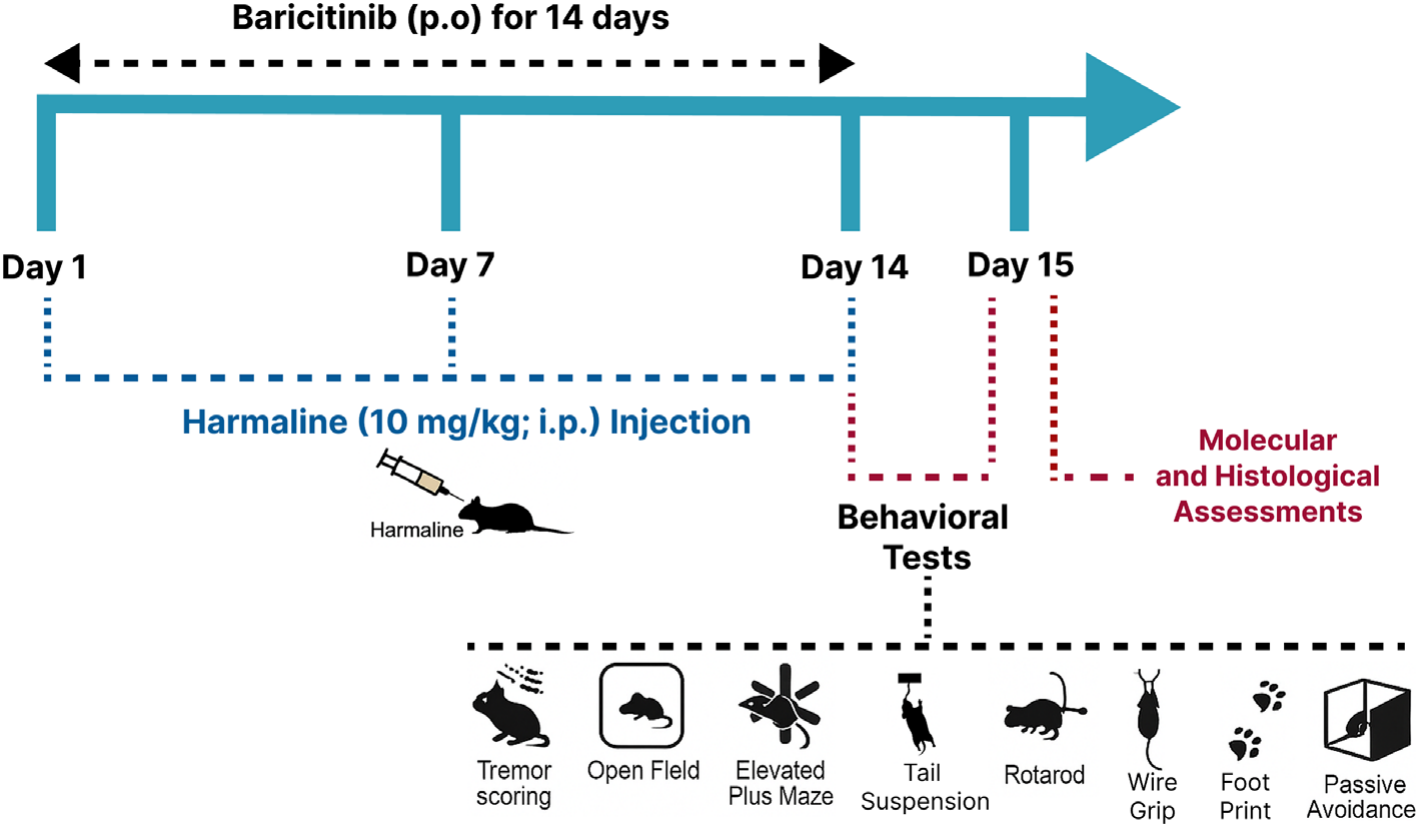
Experimental timeline and study design schematic. Mice received daily baricitinib treatment (10, 20, or 50 mg/kg) via oral gavage for 14 consecutive days, with harmaline injections (10 mg/kg, i.p.) administered on Days 1, 7, and 14. Behavioral assessments were conducted on Days 14 and 15, followed by tissue collection on Day 15 for molecular and histopathological analyses.

Accordingly, to induce the model of ET, Harmaline hydrochloride (*Sigma‐Aldrich, USA*) was dissolved in sterile saline and administered intraperitoneally (10 mg/kg) on Days 1, 7, and 14. Baricitinib (*Nano Alvand, Iran*) was dissolved in sterile saline and administered orally (10, 20, or 50 mg/kg) via gavage once daily for 14 consecutive days. Behavioral tests were conducted on Days 14 and 15. Molecular assays and histological tests were performed on Day 15. An overview of the study timeline is provided in Figure 1.

### 2.3 Behavioral Tests

The following behavioral tests were conducted to assess motor function, locomotor activity, anxiety‐like behavior, depressive‐like behavior, and cognitive performance.

Tremor Scoring: Tremor severity was evaluated using a standardized 5‐point scoring system: 0 (no tremor), 1 (occasional mild tremor in head and neck), 2 (intermittent tremor affecting all body parts), 3 (persistent tremor in all body parts and tail), and 4 (severe tremor preventing standing or walking) [24].

Open Field Test: Locomotor activity and anxiety‐like behavior were assessed using an automated video tracking system (*Ethovision software*, *Noldus Information Technology, Wageningen, The Netherlands; version 7.1*) in a 90 × 90 × 30 cm Plexiglas arena divided into central and peripheral zones. Each mouse was placed in the center and activity was recorded for 5 min. Parameters measured included: total distance traveled, duration of mobility, average velocity, time spent in peripheral zones, and frequency of rearing and grooming behaviors [25].

Elevated Plus Maze: Anxiety‐like behavior was evaluated using a plus‐shaped maze elevated 50 cm above the floor, consisting of two open arms and two closed arms. Mice were placed in the central platform facing an open arm and behavior was recorded for 5 min. Time spent in open and closed arms and the number of entries into each arm type were measured [26].

Tail Suspension Test: Depressive‐like behavior was assessed by suspending mice by their tails from a horizontal bar 50 cm above the floor using adhesive tape placed 2 cm from the tail tip. Immobility time (absence of escape‐oriented movements) was recorded during a 6‐min session [27].

Rotarod: Motor coordination was evaluated using an accelerating rotarod apparatus (*Hugo Sachs Electronik, Germany*). After two habituation sessions, mice were placed on a rotating rod that accelerated from 10 to 60 rpm over 5 min. The latency to fall was recorded over three trials with 10‐min inter‐trial intervals, and the average was calculated [28].

Wire Grip Test: Muscle strength and grip endurance were assessed by suspending mice from a horizontal steel wire (80 cm length, 7 mm diameter) by their forepaws. The latency to fall was recorded for three trials with adequate rest periods between attempts [29].

Footprint Test: Gait pattern was analyzed by applying nontoxic paint to hind paws and allowing mice to walk along a 50 cm corridor onto white paper. Stride length (distance between consecutive steps) and hind paw stride width (distance between left and right hind paw prints) were measured in millimeters [30].

Passive Avoidance Test: Learning and memory were evaluated using a shuttle box apparatus (25 × 25 × 100 cm) consisting of illuminated and dark compartments separated by a guillotine door. The protocol included three phases: (1) Adaptation: mice were placed in the light compartment and were allowed to move freely to the dark compartment. (2) Training: 2 h later, mice received a mild foot shock (0.5 mA, 2 s, 50 Hz) immediately upon entering the dark compartment, with training repeated after 1 h if re‐entry occurred; (3) Retention: 24 h post‐training, step‐through latency (STL) and time spent in the dark compartment over 5 min were measured without shock delivery (cut‐off: 300 s) [31].

### 2.4 Tissue Collection and Molecular Assays

Following behavioral testing, mice were deeply anesthetized and decapitated. Brains were rapidly removed, cerebella dissected on ice, and stored at −80°C until analysis. Total RNA was extracted from cerebellar tissue using Trizol reagent (*Zaver Zist Azma, Iran*) according to the manufacturer's instructions. RNA quality and concentration were determined using a NanoDrop spectrophotometer (*Thermo Scientific, USA*). Following DNase treatment, cDNA was synthesized using a reverse transcription kit. Real‐time PCR was performed using SYBR Green master mix. Gene expression levels for IL‐6, TNF‐α, NF‐κB, BDNF, Lingo‐1, and NMDA receptor were normalized to GAPDH and analyzed using the 2^−ΔΔCt^ method.

### 2.5 Histopathological Analysis

For histological examination, a subset of animals (*n* = 4 per group) was investigated. Brains were postfixed, paraffin‐embedded, and sectioned at five μm thickness. Although transcardial perfusion is optimal to minimize fixation artifacts, immersion fixation was used here as a routine method for comparative morphological screening. Sections were stained with hematoxylin and eosin (H&E) for morphological assessment. Purkinje cell degeneration in the cerebellar cortex was quantified by counting normal and degenerated neurons (characterized by condensed nuclei and dark cytoplasm) in three consecutive fields at 400× magnification. Investigators were blinded to group allocation during sectioning and analysis.

### 2.6 Statistical Analysis

All analyses were performed using GraphPad Prism version 8.0 software. The Shapiro–Wilk normality test was used to verify the normality of the data. Statistical differences between groups were analyzed using one‐way analysis of variance (ANOVA) followed by Tukey's post hoc test for multiple comparisons. For nonparametric data, Kruskal‐Wallis analysis was performed, followed by Dunn's multiple comparison. Outliers were identified using Grubbs test with α = 0.05 and were subsequently excluded. The data were expressed as the mean ± standard deviation (SD) or median and interquartile range. A *p* < 0.05 was considered statistically significant.

## 3 Results

### 3.1 Baricitinib Ameliorates Harmaline‐Induced Tremor

**FIGURE 2:**
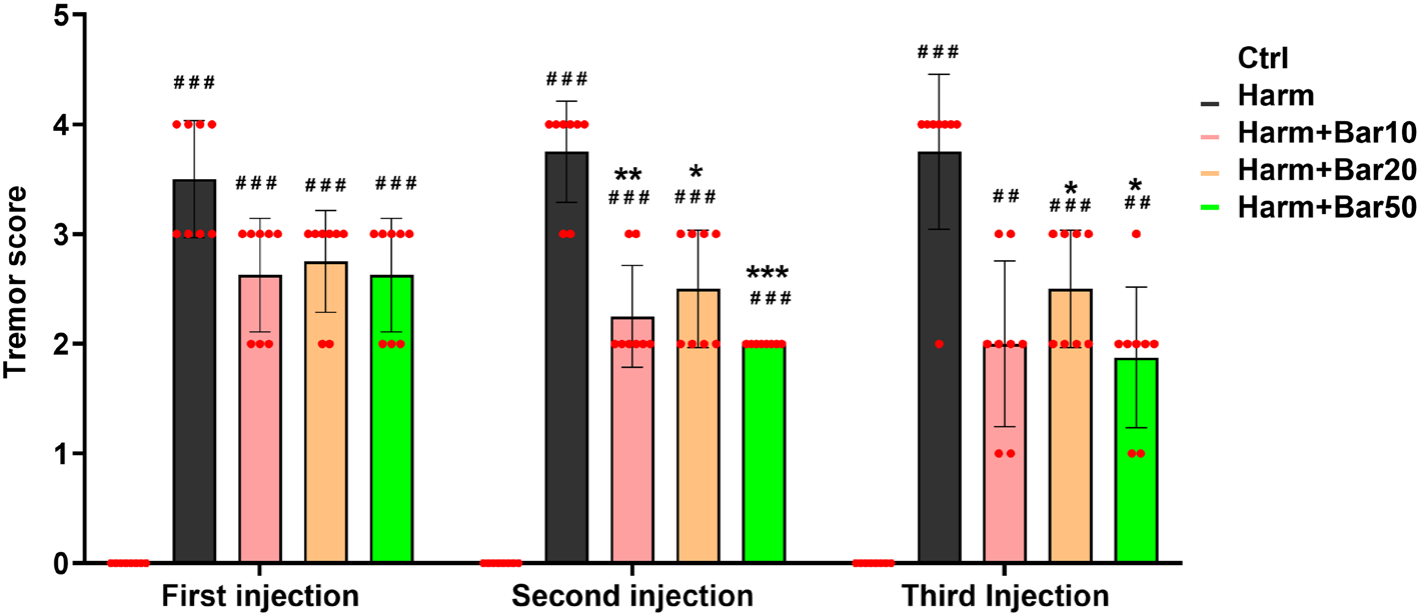
Tremor severity assessment across three harmaline injection time points. Tremor scores were evaluated using a 5‐point scale (0–4) at first, second, and third injections. Data are expressed as mean ± SEM. Ctrl: Control, Harm: Harmaline, Bar10: Baricitinib 10 mg/kg, Bar20: Baricitinib 20 mg/kg, Bar50: Baricitinib 50 mg/kg. ###*p* < 0.001 compared to control; *p < 0.05, **p < 0.01, ***p < 0.001 compared to harmaline.

Harmaline administration successfully induced significant tremor in all treatment groups compared to control animals across all three injection time points (Figure 2). Baricitinib treatment demonstrated progressive, dose‐dependent protective effects against harmaline‐induced tremor severity. Following the first injection, all baricitinib‐treated groups showed elevated tremor scores similar to the harmaline‐only group, indicating that the initial tremor induction was comparable across groups. However, by the second injection, clear therapeutic benefits emerged with baricitinib 10 mg/kg significantly reducing tremor scores (*p* < 0.01 vs. harmaline), 20 mg/kg showing moderate improvement (*p* < 0.05), and 50 mg/kg providing the most pronounced reduction (*p* < 0.001; Figure 2). Both the 20 mg/kg and 50 mg/kg doses demonstrated similar, modest improvements (*p* < 0.05 vs. harmaline for both doses) in the third injection (Figure 2).

### 3.2 Baricitinib Partially Improves Locomotor Activity and Reduces Anxiety‐Related Behaviors in the Open Field Test

**FIGURE 3:**
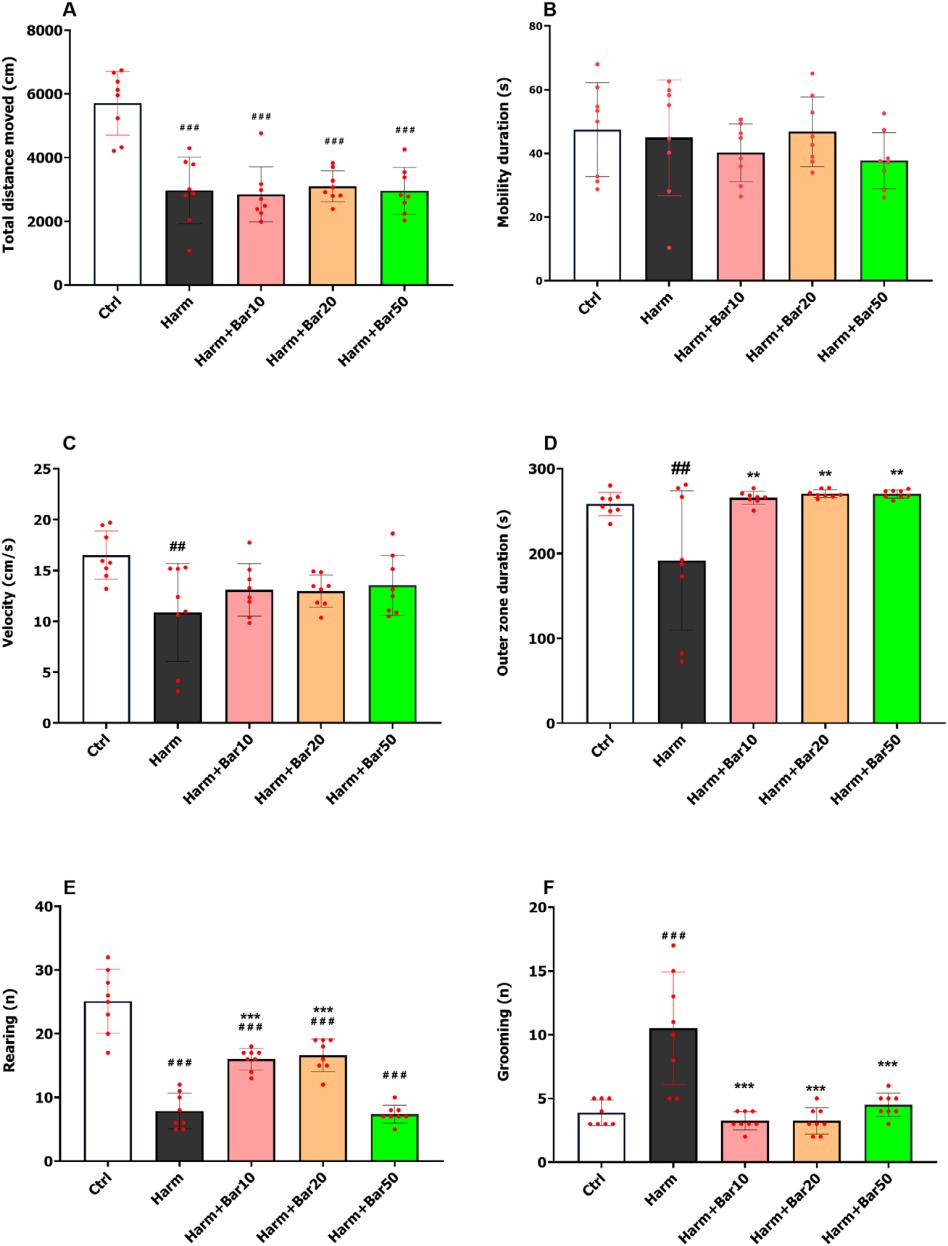
Open field assessment of locomotor activity and exploratory behaviors. (A) Total distance moved, (B) mobility duration, (C) velocity, (D) time spent in the outer zone, (E) rearing number, and (F) grooming number. Data are expressed as mean ± SEM. ##*p* < 0.01, ###*p* < 0.001 compared to control; **p < 0.01, ***p < 0.001 compared to harmaline.

Open field test analysis revealed significant effects of harmaline treatment on locomotor activity and exploratory behaviors, with baricitinib demonstrating partial protective effects. Harmaline administration significantly reduced total distance traveled compared to control animals (Figure 3A, *p* < 0.001), indicating impaired locomotor activity. All baricitinib treatment groups (10, 20, and 50 mg/kg) showed similar reductions in total distance moved compared to controls (*p* < 0.001 for all doses), suggesting that baricitinib did not restore normal locomotor activity levels. Mobility duration analysis showed no significant differences between any of the treatment groups (Figure 3B), indicating that the overall time spent in active movement was not differentially affected by the treatments. Velocity measurements revealed that harmaline significantly reduced average movement velocity (Figure 3C, *p* < 0.01 vs. control), while baricitinib‐treated groups showed numerical improvements that did not reach statistical significance but were comparable to the control group (Figure 3C). Interestingly, time spent in the outer zone was significantly reduced in the harmaline group compared to controls (Figure 3D), but all baricitinib treatment groups showed restored outer zone exploration times comparable to control levels (*p* < 0.01 vs. harmaline for all doses). Furthermore, rearing behavior was significantly decreased by harmaline treatment (Figure 3E, *p* < 0.001 vs. control), but was significantly restored by both 10 mg/kg and 20 mg/kg baricitinib (*p* < 0.001 vs. harmaline). Grooming behavior was also significantly increased by harmaline treatment (Figure 3F, *p* < 0.001 vs. control), indicative of stereotypic behavior, and this was significantly reduced by all baricitinib doses (*p* < 0.001 vs. harmaline), suggesting normalization of repetitive behaviors. These findings indicate that while baricitinib did not fully restore locomotor function, it ameliorated anxiety‐related behaviors and normalized exploratory and grooming activities in harmaline‐treated mice.

### 3.3 Baricitinib Reduces Anxiety‐Like and Depressive‐Like Behaviors

**FIGURE 4:**
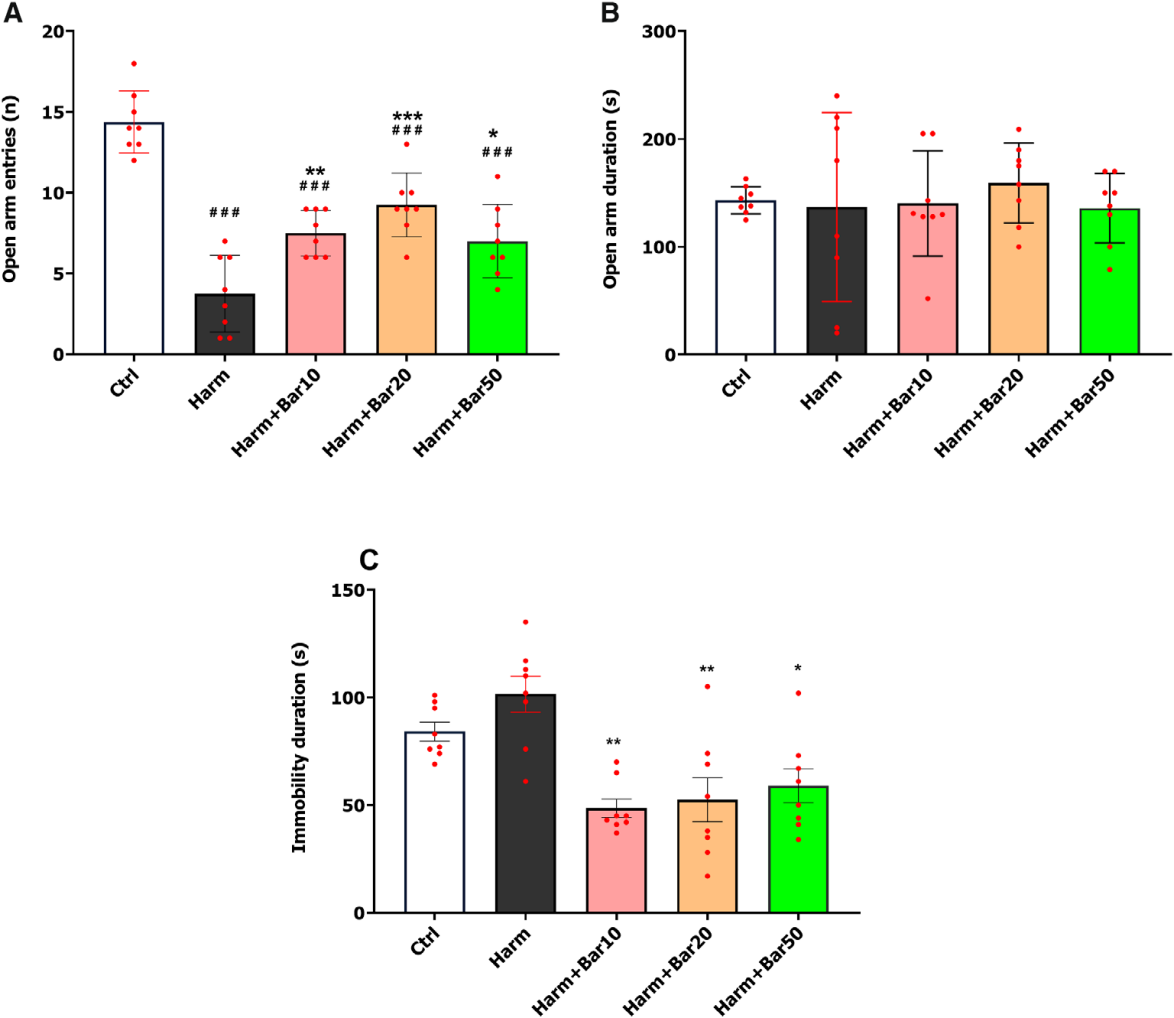
Anxiety‐like and depressive‐like behavioral assessments. (A) Open arm entries and (B) time spent in the open arms in elevated plus maze test. (C) Immobility duration in tail suspension test. Data are expressed as mean ± SEM. ###*p* < 0.001 compared to control; *p < 0.05, **p < 0.01, ***p < 0.001 compared to harmaline.

In the elevated plus maze, harmaline administration markedly reduced the number of open arm entries compared to control animals (Figure 4A, *p* < 0.001), indicating increased anxiety‐like behavior. Baricitinib treatment reversed this anxiety phenotype in 10 mg/kg (*p* < 0.01 vs. harmaline), 20 mg/kg (*p* < 0.001 vs. harmaline), and 50 mg/kg (*p* < 0.05 vs. harmaline) doses. The duration spent in open arms showed no significant differences between any treatment groups (Figure 4B). Additionally, in the tail suspension test, while harmaline alone showed a trend toward increased immobility duration compared to controls, this difference did not reach statistical significance (Figure 4C). However, all baricitinib treatment groups exhibited significantly reduced immobility times compared to the harmaline group (*p* < 0.01 for 10 mg/kg and 20 mg/kg doses, *p* < 0.05 for 50 mg/kg dose), indicating decreased depressive‐like behavior.

### 3.4 Baricitinib Shows Modest Effects on Motor Coordination and Muscle Strength

**FIGURE 5:**
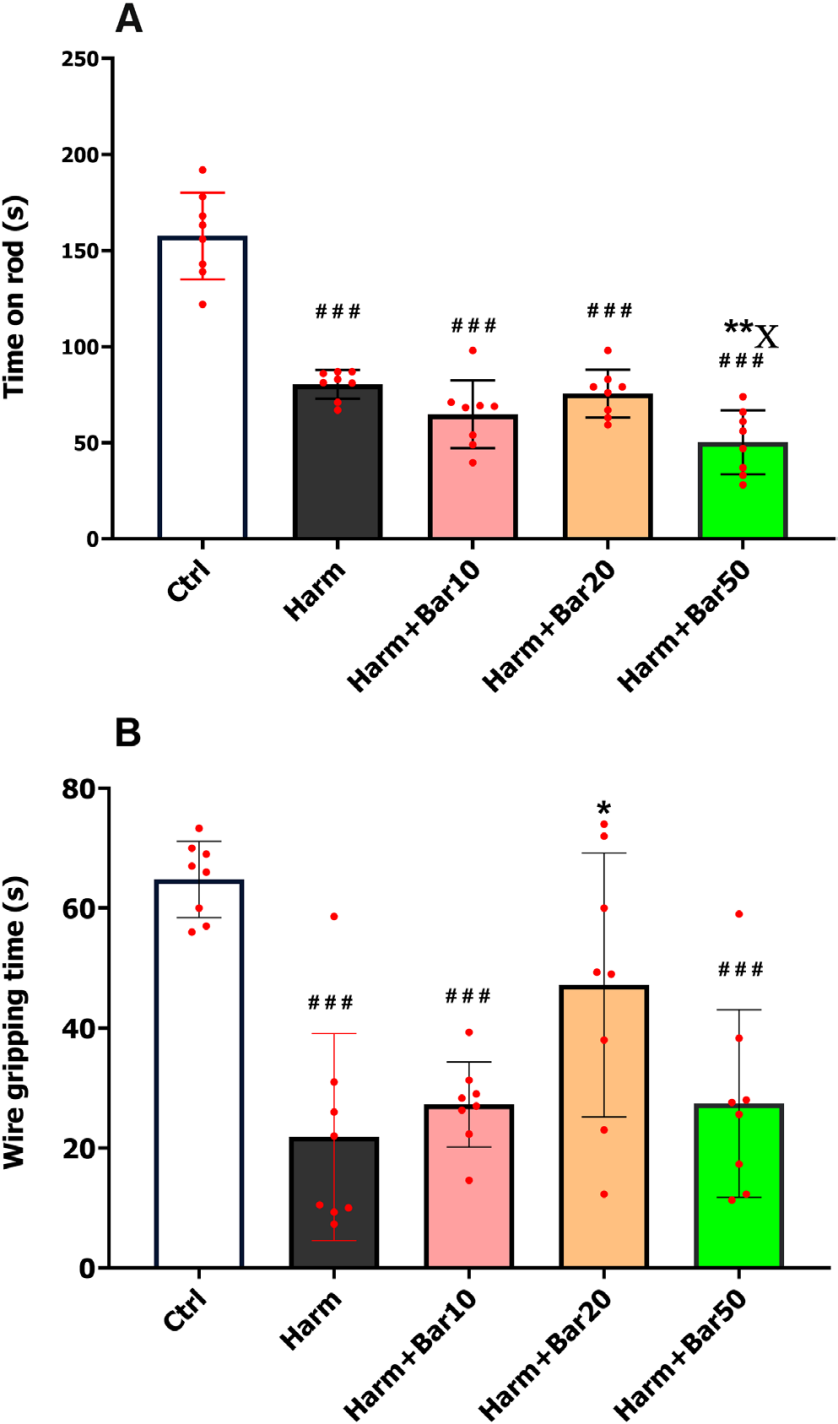
Motor coordination and muscle strength evaluations. (A) Time on rod in rotarod test and (B) wire gripping time in wire grip test. Data are expressed as mean ± SEM. ###*p* < 0.001 compared to control; *p < 0.05, **p < 0.01 compared to harmaline; ★*p* < 0.05 compared to baricitinib 20 mg/kg.

Motor coordination assessment using the rotarod test revealed that harmaline treatment significantly impaired balance and coordination compared to control animals (Figure 5A, *p* < 0.001). Baricitinib treatment did not significantly improve rotarod performance at any dose tested, as all baricitinib‐treated groups (10, 20, and 50 mg/kg) showed similar impairments compared to controls (*p* < 0.001 for all doses). Notably, the 50 mg/kg baricitinib group showed additional significant impairment compared to the harmaline‐only group (*p* < 0.01), suggesting that higher doses may negatively impact motor coordination.

Muscle strength evaluation using the wire grip test demonstrated that harmaline significantly reduced grip strength and endurance compared to controls (Figure 5B, *p* < 0.001). Interestingly, the 10 mg/kg dose of baricitinib did not significantly improve grip time compared to harmaline alone, while the 20 mg/kg dose showed significant improvement (*p* < 0.05 vs. harmaline), nearly restoring grip time to control levels. However, the 50 mg/kg dose showed impaired performance similar to the harmaline‐only group (*p* < 0.001 vs. control). These findings suggest that while moderate doses of baricitinib (20 mg/kg) may provide some benefit for muscle strength and grip endurance, higher doses do not confer additional advantages.

### 3.5 Baricitinib Does Not Improve Gait Pattern Abnormalities

**FIGURE 6:**
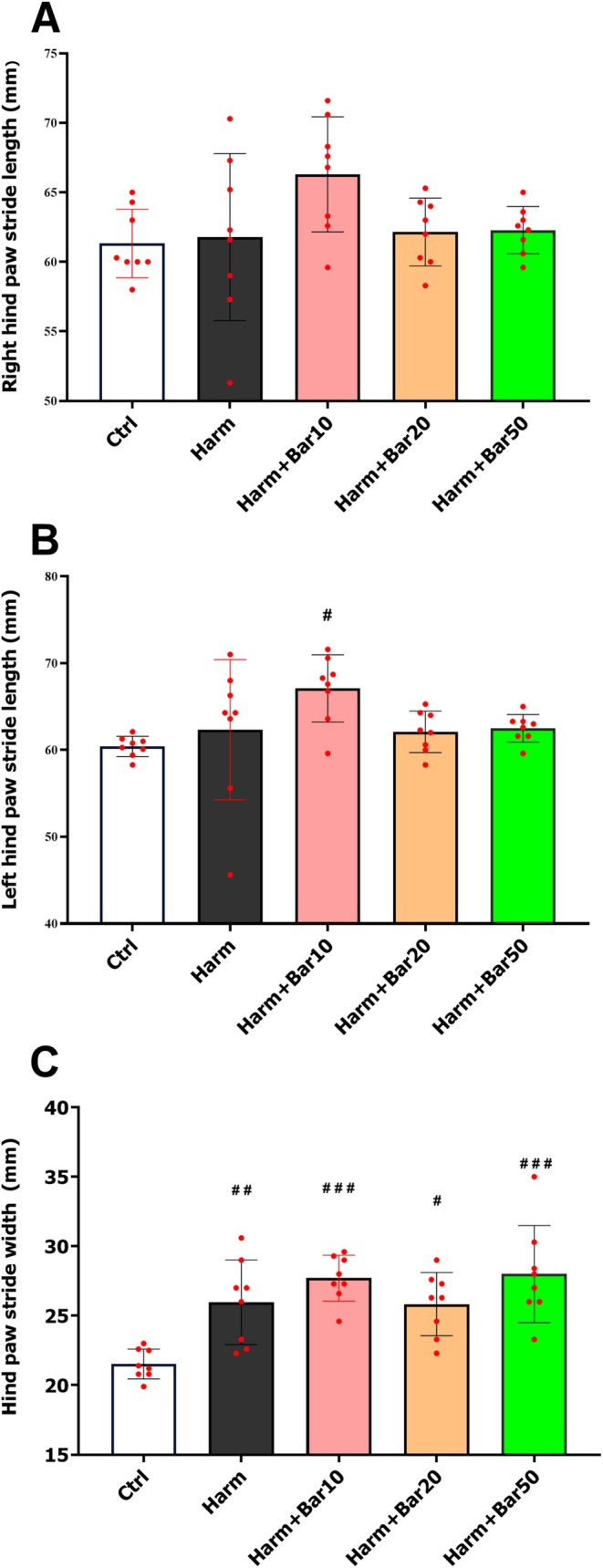
Gait pattern analysis using footprint test. (A) Right hind paw stride length, (B) left hind paw stride length, and (C) hind paw stride width measurements. Data are expressed as mean ± SEM. #*p* < 0.05, ##*p* < 0.01, ###*p* < 0.001 compared to control.

Assessment of right hind paw stride length showed no significant differences between any of the treatment groups (Figure 6A), indicating that harmaline did not substantially affect the distance between consecutive steps on the right side, and baricitinib treatment did not alter this parameter. Similarly, left hind paw stride length analysis showed minimal changes, with only the 10 mg/kg baricitinib group showing a slight but significant increase compared to controls (Figure 6B, *p* < 0.05), though this difference was not significant compared to the harmaline group. In contrast, hind paw stride width, which reflects gait stability and balance, was significantly increased in all harmaline‐treated groups compared to controls (Figure 6C). The harmaline‐only group showed increased stride width (*p* < 0.01 vs. control), indicating a wider‐based gait pattern typical of balance impairment. All baricitinib treatment groups maintained similarly increased stride widths (*p* < 0.001 for 10 mg/kg, *p* < 0.05 for 20 mg/kg, and *p* < 0.001 for 50 mg/kg vs. control), suggesting that baricitinib did not normalize the compensatory gait widening induced by harmaline. These findings indicate that harmaline primarily affects gait stability, and baricitinib treatment did not provide therapeutic benefit for normalizing this specific gait abnormality associated with tremor, with the wider‐based gait persisting across all treatment groups.

### 3.6 Passive Avoidance Outcomes Are Altered by Baricitinib

**FIGURE 7:**
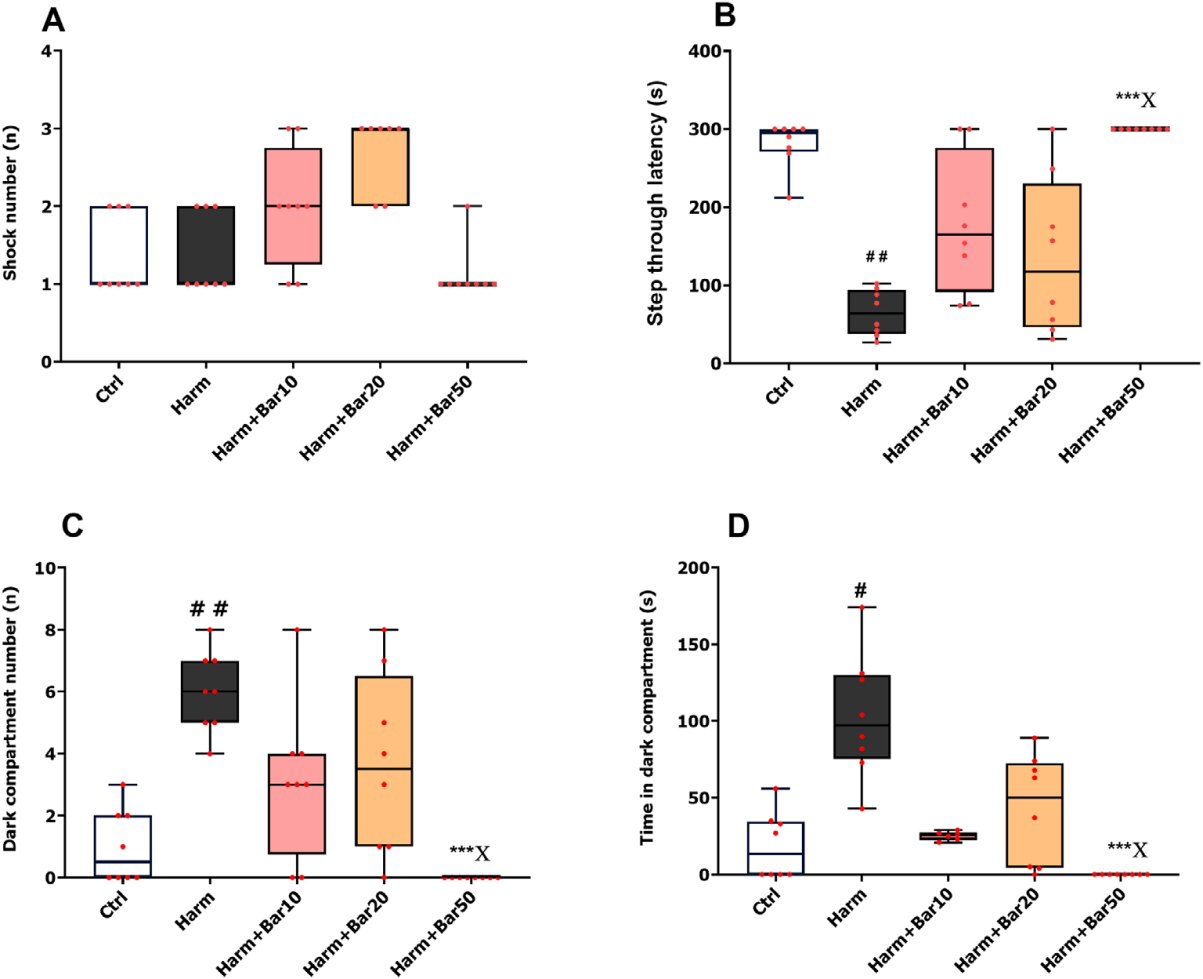
Passive avoidance learning and memory assessment. (A) Number of shocks required during training, (B) step‐through latency, (C) dark compartment entries, and (D) time spent in dark compartment. Data for all measures in this experiment were not normally distributed and are represented as medians with interquartile ranges as a box and maxima/minima as whiskers. #*p* < 0.05, ##*p* < 0.01 compared to control; ***p < 0.001 compared to harmaline; X *p* < 0.05 compared to baricitinib 20 mg/kg. *n* = 8, except for the Harm+Bar20 group in panel A and Harm+Bar10 group in Panel D, where *n* = 7 due to the exclusion of outliers.

Passive avoidance learning assessment revealed that harmaline treatment impaired memory consolidation and retention, while baricitinib demonstrated protective effects on cognitive function. During the training phase, the number of shocks required for learning did not differ significantly between the groups (Figure 7A). However, step‐through latency analysis during the retention test showed significant memory impairment in the harmaline group compared to controls (Figure 7B, *p* < 0.01), with reduced latency to enter the dark compartment indicating poor memory retention. Baricitinib 10 and 20 mg/kg groups showed comparable step‐through latency with the control group, while 50 mg/kg demonstrated significantly longer step‐through latency compared to the harmaline group (*p* < 0.001). Furthermore, the number of entries into the dark compartment was significantly increased in the harmaline group (Figure 7C, *p* < 0.01 vs. control), suggesting impaired learning. Both the 10 mg/kg and 20 mg/kg baricitinib groups showed comparable dark compartment entries with the control group, and the 50 mg/kg group showed significantly lower entries (*p* < 0.001 vs. harmaline). Similarly, time spent in the dark compartment was significantly increased in harmaline‐treated animals (Figure 7D, *p* < 0.05 vs. control). The 50 mg/kg baricitinib group significantly reduced time in the dark compartment compared to harmaline (p < 0.001). However, the enhanced performance observed at the 50 mg/kg dose should be interpreted with caution, as the concurrent motor coordination deficits in this group (Section 3.4) indicate that nonspecific motor effects may have contributed to the passive avoidance results.

### 3.7 Baricitinib Modulates Cerebellar Gene Expression of Inflammatory Markers and Neurotrophic Factors

**FIGURE 8:**
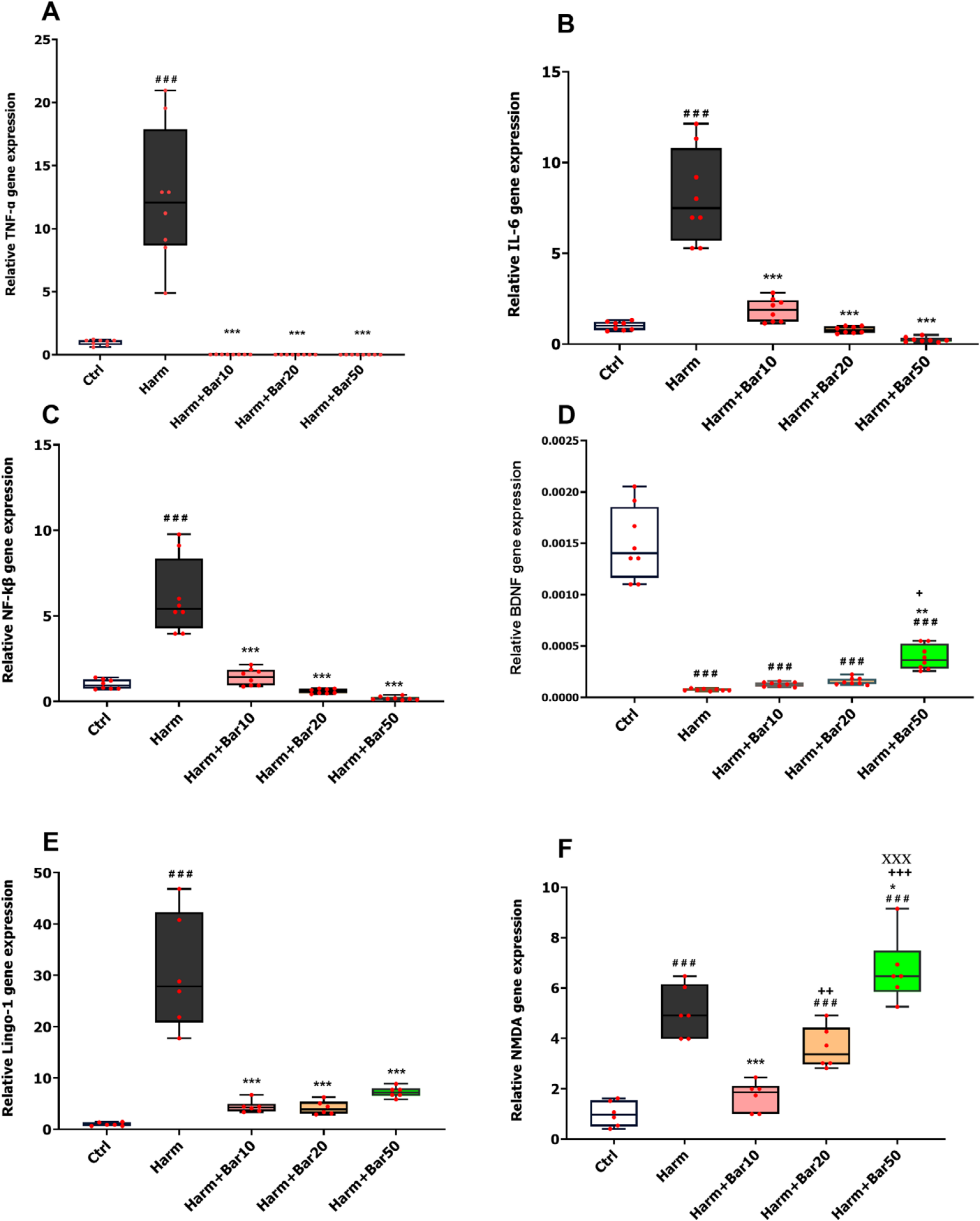
Cerebellar gene expression analysis of inflammatory markers and neurotrophic factors. Relative gene expression levels of (A) TNF‐α, (B) IL‐6, (C) NF‐κB, (D) BDNF, (E) Lingo‐1, and (F) NMDA receptor. Data for all measures in this experiment were not normally distributed and are represented as medians with interquartile ranges as a box and maxima/minima as whiskers. ###*p* < 0.001 compared to control; *p < 0.05, ***p < 0.001 compared to harmaline; XXX*p* < 0.001 compared to baricitinib 20 mg/kg; ++*p* < 0.01, +++*p* < 0.001 compared to baricitinib 10 mg/kg.

Molecular analysis of cerebellar tissue revealed significant alterations in inflammatory and neurotrophic gene expression following harmaline treatment, with baricitinib demonstrating potent anti‐inflammatory effects. Harmaline administration markedly upregulated the expression of pro‐inflammatory cytokines compared to controls. TNF‐α gene expression was significantly increased in the harmaline group (Figure 8A, *p* < 0.001), and this elevation was completely normalized by all doses of baricitinib treatment (*p* < 0.001 for all doses vs. harmaline). Similarly, IL‐6 expression was significantly elevated by harmaline (Figure 8B, *p* < 0.001) and was effectively suppressed by all baricitinib doses (*p* < 0.001 for all doses vs. harmaline), with treated groups showing expression levels comparable to or below control values. Furthermore, NF‐κB, a key transcriptional regulator of inflammatory responses, showed substantial upregulation following harmaline treatment (Figure 8C, *p* < 0.001 vs. control). All baricitinib doses significantly reduced NF‐κB expression compared to the harmaline group (*p* < 0.001 for all doses), demonstrating effective inhibition of this critical inflammatory pathway.

On the other hand, BDNF expression was significantly reduced by harmaline treatment (Figure 8D, *p* < 0.001 vs. control). Baricitinib treatment showed dose‐dependent restoration of BDNF levels, with the 50 mg/kg dose providing significant improvement compared to harmaline (*p* < 0.05). Additionally, Lingo‐1 expression was dramatically increased by harmaline treatment (Figure 8E, *p* < 0.001 vs. control), consistent with its role in inhibiting myelination and neuronal repair. All baricitinib doses effectively suppressed Lingo‐1 expression (*p* < 0.001 for all doses vs. harmaline), suggesting potential benefits for cerebellar repair mechanisms. Interestingly, NMDA receptor expression showed that harmaline significantly increased expression (Figure 8F, *p* < 0.001 vs. control), while baricitinib 10 mg/kg normalized the levels (*p* < 0.001 vs. harmaline) but no such effect was seen at higher doses.

### 3.8 Histopathological Findings Suggest Reduced Harmaline‐Associated Cerebellar Damage After Baricitinib Treatment

**FIGURE 9:**
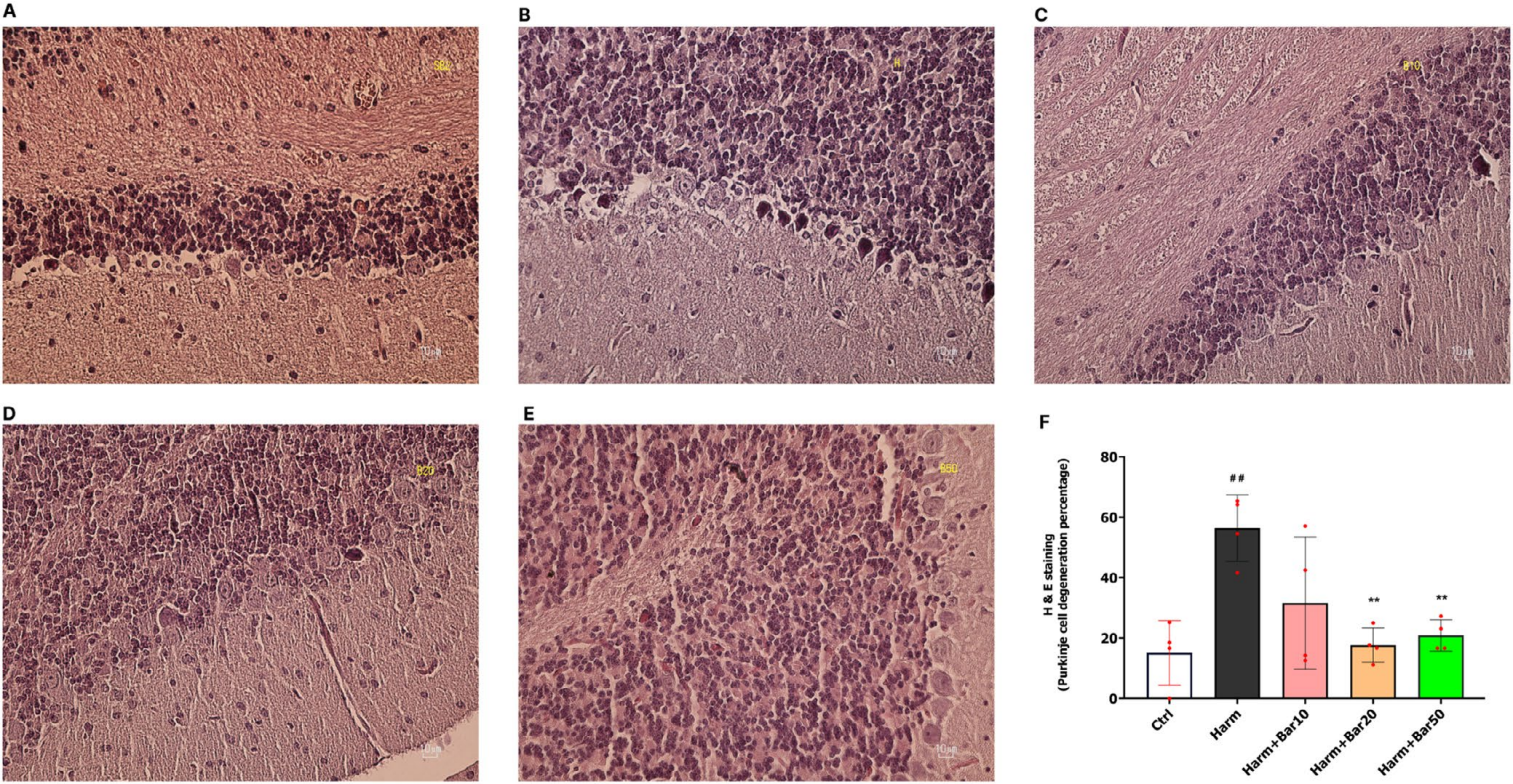
Histopathological analysis of cerebellar tissue. Representative photomicrographs of cerebellar cortex from (A) control, (B) harmaline, (C) harmaline + baricitinib 10 mg/kg, (D) harmaline + baricitinib 20 mg/kg, and (E) harmaline + baricitinib 50 mg/kg groups (H&E staining, 400× magnification). (F) Quantitative analysis of Purkinje cell degeneration percentage. Data are expressed as mean ± SEM. ##*p* < 0.01 compared to control; **p < 0.01 compared to harmaline.

Histopathological examination of cerebellar tissue suggested a reduction in harmaline‐associated morphological damage of baricitinib. Representative photomicrographs demonstrate the cerebellar cortex architecture across treatment groups (Figure 9A–E). Control cerebellum (Figure 9A) showed normal histological architecture with well‐organized Purkinje cell layer and intact molecular and granular layers. Harmaline treatment (Figure 9B) resulted in substantial cerebellar pathology, characterized by Purkinje cell degeneration, disorganized cellular layers, and increased tissue damage. Quantitative analysis of Purkinje cell degeneration confirmed these histological observations (Figure 9F). Harmaline treatment significantly increased the percentage of degenerated Purkinje cells compared to controls (*p* < 0.01). The 20 mg/kg and 50 mg/kg doses demonstrated the most pronounced neuroprotective effects (*p* < 0.01 for both vs. harmaline), reducing degeneration to levels comparable to control animals.

## 4 Discussion

The present study demonstrates for the first time that baricitinib, a selective JAK1/2 inhibitor, provides significant therapeutic benefits in the harmaline‐induced model of ET. Overall, our findings reveal that baricitinib treatment ameliorates tremor severity, improves anxiety‐related and depressive‐like behaviors, enhances cognitive performance, and provides neuroprotection through modulation of neuroinflammatory pathways in the cerebellum. These results support the growing evidence for neuroinflammation as a key pathophysiological mechanism in ET and highlight the therapeutic potential of JAK/STAT pathway inhibition in movement disorders.

The most prominent finding of our study was the significant reduction in harmaline‐induced tremor severity with baricitinib treatment. Our results, showing that baricitinib treatment reduced tremor scores, particularly by Day 7 at all doses and persisting through Day 14 for the 20 and 50 mg/kg doses, support the inflammatory hypothesis of ET pathogenesis. This hypothesis posits that sustained neuroinflammation contributes to the perpetuation and progression of tremor symptoms. We have previously demonstrated that harmaline injection results in overexpression of inflammatory markers in the animal model of ET [7]. Amelioration of tremor severity by baricitinib, a potent JAK1/2 inhibitor, suggests that targeting the JAK/STAT signaling pathway might interrupt the inflammatory cascades, such as the observed upregulation of TNF‐α, IL‐6, and NF‐κB that drive the abnormal neuronal activity in the cerebellum, thereby reducing cerebellar neurodegeneration and mitigating the abnormal neuronal excitability within the olivocerebellar pathway that underlies tremor generation.

Several recent studies have demonstrated the anti‐inflammatory role of baricitinib, beyond its classical application in rheumatoid arthritis. A recent study by Çakır et al. showed that baricitinib resulted in reducing inflammatory markers in serum and kidney in a rat model of sepsis‐induced kidney injury [32]. The drug has also shown efficacy in targeting chronic metabolic inflammation (i.e., metainflammation) [33], and has been shown to block TGF‐β1 pathway to repress myocardial fibrosis [34]. These findings reinforce the rationale for the therapeutic utility of JAK/STAT inhibition as a targeted anti‐inflammatory strategy in diseases, including ET.

While baricitinib showed promising improvement in tremor scores, the motor function revealed a more complex picture. In the open field test, baricitinib‐treated groups showed persistent reductions in total distance traveled and failed to restore normal locomotor activity levels, suggesting that the drug's benefits may be specific to tremor‐related circuits rather than providing broader motor enhancement. The rotarod test results further support this interpretation. This finding suggests that while JAK/STAT pathway inhibition effectively modulates tremor‐generating mechanisms, it may not address all aspects of motor dysfunction in the harmaline model. Moreover, the dose‐dependent impairment observed with higher baricitinib doses on the rotarod may reflect non‐specific effects at higher concentrations or suggest that optimal dosing requires careful titration to balance therapeutic benefits with potential side effects. On the other hand, the wire grip test revealed that moderate doses of baricitinib (20 mg/kg) provided some benefit for muscle strength and endurance, nearly restoring grip time to control levels. This finding suggests that baricitinib may have differential effects on various aspects of motor function, with potential benefits for muscle performance that could be relevant to the functional disability experienced by ET patients.

It is important to highlight that while baricitinib showed benefits in reducing tremor severity, its effects on motor function were selective. The drug did not address the underlying gait abnormalities associated with tremor, as indicated by the consistently increased hind paw stride width observed in all treatment groups. This finding suggests that the mechanisms responsible for generating tremor in this model may be distinct from those that lead to compensatory gait widening. Alternatively, it could indicate that the anti‐inflammatory properties of baricitinib are insufficient to reverse this specific aspect of tremor‐related cerebellar dysfunction.

Furthermore, the present results reveal a nonlinear, inverted U‐shaped dose–response relationship for baricitinib in this model. While lower doses (10 and 20 mg/kg) effectively reduced tremor, modulated inflammatory and neurotrophic markers, and improved affective behaviors, the highest dose (50 mg/kg) was associated with a loss of efficacy in regulating certain molecular targets (e.g., NMDA receptor) and induced significant motor coordination deficits (Rotarod). This pattern suggests a narrow therapeutic window and supports the hypothesis of a toxicity threshold, beyond which adverse effects on motor and possibly neuronal function may prevail. Thus, 50 mg/kg may represent a supratherapeutic dose in this model, and its effects should not be interpreted simply as enhanced therapeutic potency.

Another important finding of our study was baricitinib's ability to ameliorate anxiety‐related and depressive‐like behaviors induced by harmaline treatment. In the elevated plus maze, all doses of baricitinib significantly increased open arm entries, indicating reduced anxiety‐like behavior. Moreover, the normalization of time spent in the outer zone across all baricitinib doses in the open field test also suggests restoration of exploratory behavior. Additionally, the restoration of rearing behavior and reduction in excessive grooming provide further evidence for baricitinib's ability to normalize anxiety‐related and stereotypic behaviors. This anxiolytic effect is particularly relevant given that anxiety disorders are highly prevalent in ET patients. For instance, a study has shown that anxiety disorders were significantly higher in ET patients compared to controls (66% vs. 18%), which was also associated with higher rates of sleep disturbances and fatigue [35]. The tail suspension test results also revealed significant antidepressant‐like effects of baricitinib, with all doses reducing immobility time compared to harmaline treatment. This finding is particularly important given the high prevalence of depression in ET patients, which is shown to affect more than 30% of individuals with the condition [36].

The anxiolytic and antidepressant‐like effects of baricitinib may be mediated through its anti‐inflammatory properties, as mounting evidence suggests that neuroinflammation plays a crucial role in the pathogenesis of anxiety and depression [37, 38]. The reduction in pro‐inflammatory cytokines such as TNF‐α and IL‐6, which we observed in our molecular analyses, likely contributes to these mood‐enhancing effects. These findings suggest that baricitinib's benefits extend beyond motor symptoms to encompass the broader spectrum of neuropsychiatric manifestations associated with ET, and this is clinically significant, as the management of non‐motor symptoms in ET patients is often challenging and requires multimodal therapeutic approaches [39, 40].

The passive avoidance test showed that baricitinib‐treated mice, particularly at 50 mg/kg, exhibited increased step‐through latency and reduced dark‐compartment exploration compared with the harmaline group. However, these findings should not be interpreted as definitive evidence of cognitive enhancement. Passive avoidance performance depends not only on memory retention but also on locomotor ability, exploratory drive, anxiety‐like behavior, and the capacity to initiate movement. In our experimental schedule, rotarod and wire grip performance were assessed on Day 14, close to the final harmaline challenge, whereas the passive avoidance retention trial was performed on Day 15, 24 h after training and after the expected acute tremorgenic effects of harmaline had largely subsided. This timing reduces the likelihood that acute harmaline‐induced motor impairment fully explains the passive avoidance findings. Nevertheless, it does not exclude residual motor dysfunction, altered exploratory behavior, or possible high‐dose adverse effects of baricitinib. Because the 50 mg/kg dose was associated with impaired rotarod performance, noncognitive factors such as motor impairment or possible high‐dose adverse effects may have contributed to the apparent improvement in passive avoidance outcomes. Future studies using motor‐independent cognitive tasks, such as novel object recognition, or electrophysiological assessments of synaptic plasticity are required to determine whether baricitinib exerts genuine cognitive benefits in the harmaline model.

Cognitive impairment is increasingly recognized as a component of ET, with patients showing deficits in executive function, working memory, and information processing speed [41, 42, 43]. The cognitive enhancing effects of baricitinib may be mediated through multiple mechanisms. As we discuss later, the restoration of BDNF expression, which we observed in our molecular analyses, likely contributes to these cognitive benefits. BDNF is a crucial neurotrophic factor that supports synaptic plasticity, neuronal survival, and cognitive function [44, 45]. The harmaline‐induced reduction in BDNF expression and its restoration by baricitinib treatment suggest that JAK/STAT pathway inhibition may promote neuroplasticity and cognitive resilience.

Overall, our molecular analyses provide evidence for the anti‐inflammatory mechanisms underlying baricitinib's therapeutic effects. The dramatic reduction in pro‐inflammatory cytokines TNF‐α and IL‐6 across all doses demonstrates the drug's potent anti‐inflammatory properties in the cerebellum. These cytokines play crucial roles in neuroinflammation and have been implicated in the pathogenesis of various movement disorders [46, 47, 48]. Additionally, the suppression of NF‐κB expression was noteworthy, as this transcription factor serves as a master regulator of inflammatory gene expression. NF‐κB activation leads to the transcriptional upregulation of numerous pro‐inflammatory mediators, creating a self‐perpetuating cycle of neuroinflammation [49, 50]. The ability of baricitinib to suppress NF‐κB expression suggests that it interrupts this inflammatory cascade at a fundamental level, potentially preventing the chronic neuroinflammation that contributes to ET progression.

Notably, our findings regarding the modulation of Lingo‐1 expression represent another important mechanism of baricitinib's neuroprotective effects. Lingo‐1 is a negative regulator of myelination and synaptic plasticity that is upregulated in ET patients' cerebellar tissue [12, 51]. We have also previously shown that harmaline injection to induce an animal model of ET also results in overexpression of Lingo‐1 [7, 13]. The reduction in Lingo‐1 expression achieved by baricitinib treatment suggests that the drug may promote cerebellar repair mechanisms and restore normal synaptic function. This finding is of importance considering the emerging evidence for oligodendrocyte dysfunction and myelin abnormalities in ET pathogenesis [52]. Furthermore, the modulation of NMDA receptor expression provides additional mechanistic insight into baricitinib's mechanisms of action. NMDA receptors have crucial roles in synaptic plasticity and motor learning [53, 54]. Overexpression of these receptors could result in disruption of motor learning [55]. Inhibition of this overexpression by baricitinib at low doses might suggest that the drug may help restore glutamatergic signaling balance in the cerebellum. However, the lack of effect at higher doses indicates that this mechanism may be dose‐dependent and requires careful optimization.

Finally, the histopathological analyses validate baricitinib's neuroprotective properties. We observed a significant reduction in Purkinje cell degeneration with baricitinib treatment, particularly at moderate to high doses, which demonstrates the drug's ability to preserve cerebellar architecture and prevent neuronal loss. Purkinje cell pathology is generally a hallmark feature of ET, with postmortem studies and neuroimaging findings revealing structural abnormalities in ET patients' cerebella [56, 57]. The combined anti‐inflammatory and neurotrophic mechanisms of baricitinib may have provided a favorable microenvironment for neuronal survival and function.

Taken together, the findings of our study have several important clinical implications for ET management. The demonstration that JAK/STAT pathway inhibition provides therapeutic benefits in an established ET model suggests that this pathway represents a novel therapeutic target for the condition. Given the limited efficacy and significant side effects of current ET therapeutics, the identification of new treatment targets is crucial for improving patient outcomes. Moreover, the broad spectrum of benefits observed with baricitinib treatment, encompassing motor symptoms, anxiety, depression, and cognitive function, suggests that the drug could address the multifaceted nature of ET. Current treatments primarily focus on motor symptoms, leaving many patients with inadequately managed non‐motor manifestations. The ability of baricitinib to simultaneously improve multiple symptom domains could represent an advancement in ET therapeutics which warrants more research. The established safety profile of baricitinib in humans provides a pathway for clinical translation. The drug is already approved for the treatment of rheumatoid arthritis and alopecia areata [19, 58], and this existing clinical experience could facilitate the rapid translation of our preclinical findings to human studies.

Several limitations of our study should be acknowledged. The harmaline model, while well‐validated, represents an acute pharmacological model of tremor that may not fully recapitulate all aspects of human ET pathophysiology. Future studies using genetic or chronic models of ET would help validate our findings and provide additional insight into baricitinib's therapeutic potential. Furthermore, our study focused on short‐term treatment effects, and longer‐term studies are needed to assess the durability of baricitinib's benefits and potential for disease modification. Additionally, dose–response optimization studies would help identify the most effective dosing regimens for different symptom domains. Finally, while our molecular and histological analyses provide important mechanistic insights, additional studies investigating the temporal dynamics of inflammatory changes and the relationship between molecular alterations and behavioral outcomes would enhance our understanding of baricitinib's mechanisms of action. Several additional limitations should be acknowledged. First, the interpretation of cognitive enhancement at the highest dose (50 mg/kg) is limited by concurrent motor deficits, highlighting the need for future studies to employ motor‐independent cognitive assessments (e.g., novel object recognition) or electrophysiological measures of synaptic plasticity (e.g., LTP in brain slices). Second, the histopathological analysis, while suggestive, has methodological constraints. The use of immersion fixation carries a risk of ‘dark neuron’ artifact, and our 2D counting method is non‐stereological. Future work should utilize transcardial perfusion, unbiased stereological counting, and automated image analysis to confirm neuroprotection with greater rigor. Finally, the observed inverted U‐shaped dose–response underscores the importance of dose optimization in translational studies. Future research directions should include investigation of baricitinib's effects in animal models of other movement disorders, assessment of combination therapies with existing ET treatments, and exploration of biomarkers that could guide patient selection and treatment monitoring in clinical trials.

## 5 Conclusions

In conclusion, our study demonstrates that baricitinib provides significant therapeutic benefits in the harmaline‐induced ET model through modulation of neuroinflammatory pathways, improving tremor severity, affective behaviors, and cognitive function. Its effects on motor function, however, were specific to tremor reduction and did not extend to normalization of tremor‐related gait abnormality. The drug's ability to simultaneously improve tremor severity, anxiety‐related and depressive‐like behaviors, cognitive function, and cerebellar pathology suggests that JAK/STAT pathway inhibition might represent a promising therapeutic approach for ET.

## Author Contributions

**Mehran Ilaghi:** conceptualization, methodology, investigation, writing – original draft. **Leila Bagherzadeh:** conceptualization, supervision, writing – review and editing. **Sina Elyasifar:** conceptualization, methodology, investigation, data curation. **Fatemeh Shahsavari:** conceptualization, methodology, data curation, investigation. **Mohammad Shabani:** conceptualization, formal analysis, resources, supervision, funding acquisition, writing – review and editing. **Zeynab Pirmoradi:** conceptualization, methodology, formal analysis, supervision, project administration, writing – review and editing. **Monavareh Soti:** conceptualization, methodology, investigation, data curation.

## Funding

Funding for this study was provided by Kerman University of Medical Sciences (Grant no. 403001081).

## Ethics Statement

The experimental procedures were approved by the Ethics Committee of Kerman University of Medical Sciences under ethics code of IR.KMU.AEC.1404.024.

## Consent

The authors have nothing to report.

## Conflicts of Interest

The authors declare no conflicts of interest.

## Data Availability

The data that support the findings of this study are available from the corresponding author upon reasonable request.
